# Zika virus replication in the mosquito *Culex quinquefasciatus* in Brazil

**DOI:** 10.1038/emi.2017.59

**Published:** 2017-08-09

**Authors:** Duschinka RD Guedes, Marcelo HS Paiva, Mariana MA Donato, Priscilla P Barbosa, Larissa Krokovsky, Sura W dos S Rocha, Karina LA Saraiva, Mônica M Crespo, Tatiana MT Rezende, Gabriel L Wallau, Rosângela MR Barbosa, Cláudia MF Oliveira, Maria AV Melo-Santos, Lindomar Pena, Marli T Cordeiro, Rafael F de O Franca, André LS de Oliveira, Christina A Peixoto, Walter S Leal, Constância FJ Ayres

**Affiliations:** 1 Departamento de Entomologia, Instituto Aggeu Magalhães, Fundação Oswaldo Cruz (Fiocruz), Recife 50760-420, Brazil; 2 Núcleo de Ciências da Vida, Centro Acadêmico do Agreste, Universidade Federal de Pernambuco, Caruaru 55002-970, Brazil; 3 Laboratório de Virologia e Terapia Experimental, Instituto Aggeu Magalhães, Fundação Oswaldo Cruz (Fiocruz), Recife 50670-420, Brazil; 4 Núcleo de Estatística e Geoprocessamento, Instituto Aggeu Magalhães, Fundação Oswaldo Cruz (Fiocruz), Recife 50670-420, Brazil; 5 Department of Molecular and Cellular Biology, University of California-Davis, Davis, CA 95616, USA

**Keywords:** *Aedes*, *Culex*, microcephaly, vector competence, Zika

## Abstract

Zika virus (ZIKV) is a flavivirus that has recently been associated with an increased incidence of neonatal microcephaly and other neurological disorders. The virus is primarily transmitted by mosquito bite, although other routes of infection have been implicated in some cases. The *Aedes aegypti* mosquito is considered to be the main vector to humans worldwide; however, there is evidence that other mosquito species, including *Culex quinquefasciatus*, transmit the virus. To test the potential of *Cx. quinquefasciatus* to transmit ZIKV, we experimentally compared the vector competence of laboratory-reared *Ae. aegypti* and *Cx. quinquefasciatus*. Interestingly, we were able to detect the presence of ZIKV in the midgut, salivary glands and saliva of artificially fed *Cx. quinquefasciatus*. In addition, we collected ZIKV-infected *Cx. quinquefasciatus* from urban areas with high microcephaly incidence in Recife, Brazil. Corroborating our experimental data from artificially fed mosquitoes, ZIKV was isolated from field-caught *Cx. quinquefasciatus*, and its genome was partially sequenced. Collectively, these findings indicate that there may be a wider range of ZIKV vectors than anticipated.

## INTRODUCTION

Zika is classically considered a mild disease whose symptoms include fever, joint pain, rash and, in some cases, conjunctivitis.^1^ However, the recent Zika outbreak in Brazil has been associated with an increased incidence of neonatal microcephaly and neurological disorders.^2, 3^ Zika virus (ZIKV) is a poorly understood, small, enveloped RNA virus with ssRNA (+) belonging to the family Flaviviridae. It was first isolated in April 1947 from a rhesus monkey and then, in January 1948, from the mosquito species *Aedes africanus*.^4^ Subsequently, several ZIKV strains have been isolated from *Aedes*, *Mansonia, Anopheles* and *Culex* mosquitoes.^5^

The first known Zika epidemic in an urban environment occurred in Micronesia in 2007, with ~73% of the human population on Yap Island becoming infected.^6^ Intriguingly, although many *Aedes* mosquitoes were collected in the field and evaluated for virus detection, no samples were found to be positive for ZIKV.^6^ In addition, it is important to highlight that *Ae. aegypti* is absent from most islands in the Micronesian archipelago and is rare on the islands where ZIKV is present.^6, 7^

There is a global consensus among scientists and health agencies that *Ae. aegypti* and *Ae. albopictus* are the main ZIKV vectors in urban areas (WHO, 2016). This belief is partly because vector competence experiments for ZIKV were conducted exclusively for species of this genus, mainly *Ae. aegypti*, until recently.^8, 9^ In fact, previous laboratory studies^8, 10^ suggested that *Ae. aegypti* is a ZIKV vector. Recently, high rates of dissemination and transmission of the ZIKV in *Ae. aegypti* have been observed under laboratory conditions.^11^ Intriguingly, a few studies have demonstrated that *Ae. aegypti* and *Ae. albopictus* populations have low rates of ZIKV transmission^12, 13^ or none,^14, 15^ but the role of other vectors in the spread of ZIKV has been overlooked. Thus, other mosquito species, such as *Cx. quinquefasciatus*, that coexists with *Ae. aegypti* in urban areas, could contribute to ZIKV transmission.^16^ The literature is dichotomous regarding *Culex* vectors. Although there are recent reports showing that *Culex* is not a ZIKV vector,^17, 18, 19, 20, 21, 22^ a recent paper demonstrated that *Cx. quinquefasciatus* collected in urban areas in China were able to be infected with a local ZIKV strain and then transmit it to mice.^23^ These controversial results are expected and could be due to differences in the combination of mosquito and virus genotypes used in artificial blood-feeding assays, as previous studies have also shown negative results for the main vector *Ae. aegypti*.^13, 15^ Diagne *et al.*^15^ reported that Dakar and Kedougou (Senegal) populations of the yellow fever mosquito did not transmit six different ZIKV strains, including the MR766 strain, which was first isolated from a sentinel monkey in Uganda. In an evident contrast, Wegner-Lucarelli *et al.*^24^ found that *Ae. aegypti* from Poza Rica (Mexico) is a competent vector of the MR766 ZIKV strain. These are the first two studies that used the same strain of the virus *vis-à-vis* the same mosquito species. Therefore, it is reasonable to conclude that the genetic variability of mosquito species from different geographical origins might account for this apparent discrepancy in reported data.^25^ Here, we report data that support the idea that in Recife, northeast Brazil, *Cx. quinquefasciatus*, the southern house mosquito in tropical and subtropical areas, is a potential ZIKV vector. We performed mosquito vector competence assays under laboratory conditions, comparing both *Ae. aegypti* and *Cx. quinquefasciatus* using different virus doses. ZIKV was detected in the salivary glands and in the saliva of artificially fed *Cx. quinquefasciatus* mosquitoes. In addition to these results, ZIKV was also detected in field-caught samples of *Cx. quinquefasciatus*, which had no signs of recent blood feeding. ZIKV was isolated from these samples, and its genome was sequenced, providing the first partial genome of ZIKV obtained from *Cx. quinquefasciatus* mosquitoes. Collectively, our results suggest that this species is likely a ZIKV vector in Brazil, which has several implications for vector control strategies as well as for the understanding of the epidemiology of ZIKV.

## MATERIALS AND METHODS

### Mosquitoes

The present study was conducted using two laboratory colonies of mosquitoes and field-collected specimens of *Ae. aegypti* (F_1_–F_2_) from the Archipelago of Fernando de Noronha (FN), a district of Pernambuco State (PE). *Cx. quinquefasciatus* (formerly known as *Cx. pipiens quinquefasciatus*) mosquitoes originated from eggs (rafts) collected in Peixinhos, a neighborhood in Recife, PE, Brazil, in 2009. The *Ae. aegypti* laboratory colony (RecLab) was established with ~1000 specimens collected in Graças, a neighborhood in the Recife Metropolitan Region, and has been maintained in the insectary of Aggeu Magalhães Institute (IAM)/FIOCRUZ since 1996 under standard conditions: 26±2 °C, 65%–85% relative humidity, 12/12 light/dark cycle. More information regarding the two laboratory colonies has been described elsewhere.^26, 27^ The mosquitoes were kept in the insectary of the Department of Entomology IAM/FIOCRUZ under standard conditions described above. Larvae were maintained in plastic trays filled with potable water and were fed solely on cat food (Friskies), while adults were given access to a 10% sucrose solution until they were administered defibrinated rabbit blood infected with ZIKV.

### Virus strain

Experimental infections of mosquitoes with ZIKV were conducted using the ZIKV BRPE243/2015 strain derived from the serum of a patient with an acute maculopapular rash in Pernambuco State, Brazil, during the 2015 outbreak.^28^ This strain was named the ZIKV/*H. sapiens*/Brazil/PE243/2015 strain, according to the nomenclature described by Scheuermann,^29^ and was fully characterized (accession number KX197192.1). Following isolation, the virus was passed once on *Ae. albopictus* C6/36 cells. Viral stocks were then produced in VERO cells and stored at −80 °C until use. Prior to oral infection, the viral titer of the stock was calculated via plaque assay on VERO cells and reached 10^6^ plaque-forming units per milliliter (PFU/mL).

### Artificial feeding

Two artificial-feeding assays were conducted using a viral stock concentration of 10^6^ PFU/mL as well as a 100-fold dilution from the viral stock in each experiment. Notably, in the first artificial-feeding assay, the frozen virus sample was mixed with defibrinated rabbit blood. In the second assay, ZIKV BRPE243/2015 was first grown in VERO cells at a multiplicity of infection of 0.5 for 4–5 days. Subsequently, the cell culture flasks were frozen at −80 °C, thawed at 37 °C twice and then mixed with defibrinated rabbit blood in a 1:1 proportion. Seven- to ten-day-old female mosquitoes were starved for 18 h prior to artificial feeding. The mosquitoes were exposed to an infectious blood meal for 90 min, as described in Salazar *et al.*,^30^ with minor modifications. Briefly, infectious blood was provided in a membrane-feeding device placed on each mosquito cage. The blood feeding was maintained at 37 °C by using heat packs during the process. Fully engorged mosquito females were cold anesthetized, transferred to a new cage and maintained in the infection room for 15 days. Both assays included a control group fed on uninfected cultured cells mixed with defibrinated rabbit blood.

### RNA extraction and virus detection

Four to fifteen mosquitoes were dissected in order to collect the midguts and salivary glands at three, seven and 15 days post infection (dpi). To prevent hemolymph contamination, all midguts and salivary glands were extensively washed with phosphate-buffered saline buffer immediately after dissection and prior to tissue extraction. Tissues were individually transferred to a 1.5-mL DNAse/RNAse-free microtube containing 300 μL of mosquito diluent^31^ and were stored at −80 °C until further usage. Each tissue was ground with sterile micropestles, and RNA extraction was performed with 100 μL of the homogenate. The TRIzol method (Invitrogen, Waltham, MA, USA) was performed according to the manufacturer’s instructions with modifications as follows. Tissue homogenate (100 μL) was mixed with 200 μL of TRIzol, homogenized by vortexing for 15 s and incubated for 5 min at room temperature. Chloroform (100 μL) was added to the mixture, and homogenization was performed by shaking the tubes vigorously for 15 s by hand. The mixture was then incubated at room temperature for 2–3 min. The samples were centrifuged at 12 000*g* for 15 min at 4 °C. The aqueous phase of each sample was removed and transferred to a new tube containing 250 μL of 100% isopropanol. This mixture was incubated at room temperature for 10 min and then centrifuged at 12000*g* for 10 min at 4 °C. The supernatant was removed, and the RNA pellet was washed with 300 μL of 75% ethanol. The samples were homogenized briefly and then centrifuged at 7500*g* for 5 min at 4 °C. The supernatant was discarded, and the RNA was then air-dried for 15 min. The RNA pellet was resuspended in 30 μL of RNAse-free water. After RNA resuspension, the samples were treated with DNAse (Turbo DNase, Ambion, Foster City, CA, USA) according to the manufacturer’s protocol.

Virus detection was performed by quantitative RT-PCR (RT-qPCR, also known as real-time RT-PCR) in an ABI Prism 7500 SDS Real-Time system (Applied BioSystems, Foster City, CA, USA) using the QuantiNova Probe RT-PCR kit (Qiagen, Hilden, Germany). RT-qPCR was performed in a 20-μL reaction volume containing 5 μL of extracted RNA, 2 × QuantiNova Probe RT-PCR Master Mix, 0.2 μL of the QuantiNova Probe RT Mix, 0.1 μL of the ROX Reference Dye, 100 μM of each primer (stock) and 25 μM of the probe (stock). The primers, probe and PCR conditions were first described in Lanciotti *et al.*,^32^ and each sample was tested in duplicate. RT-qPCR cycling included a single cycle of reverse transcription for 15 min at 45 °C, followed by 5 min at 95 °C for reverse transcriptase inactivation and DNA polymerase activation, and then 45 cycles of 5 s at 95 °C and 45 s at 60 °C (annealing-extension step). The amount of viral RNA in each sample was estimated by comparing the cycle threshold values (Ct) to the standard curve for every RT-qPCR assay. The standard curve consisted of different dilutions of previously titrated ZIKV BRPE243/2015 RNA. Mosquitoes collected immediately after artificial feeding were used as positive controls, while control mosquitoes fed on uninfected blood and RT-PCR reactions containing no RNA represented negative controls. Fluorescence was analyzed at the end of the amplifications. Positive samples were used to calculate vector competence parameters, such as the infection rate (IR), which is the number of positive midguts divided by the total number of midguts tested, and the proportion of infected salivary glands (SR), which is the number of positive salivary glands divided by the total number of salivary glands tested.

### Collection of virus-infected mosquito saliva

To confirm whether the viral RNA detected by RT-qPCR within the salivary glands was infective and could be released during blood-feeding meals, we assayed ZIKV from saliva samples. From the 8th to 14th dpi, mosquitoes from each group were exposed to honey-soaked Flinders Technology Associates (FTA) Classic Cards (Whatman, Maidstone, UK) placed on the top of the cages to collect mosquito saliva. Each FTA card was prepared in a sterilized Petri dish and soaked in ~10 g of anti-bacterial honey (Manuka Honey Blend, Arataki Honey Ltd, Havelock North, Hastings, New Zealand) and 1 mL of blue food dye (Soft Gel Mix) for 2 h. The blue food dye was used to determine whether the mosquitoes had fed on the FTA cards. After 24 h of exposure, each card was placed in a 15-mL falcon tube and stored at −80 °C until further use. To extract the RNA, the cards were placed individually in 2-mL microtubes with 600 μL of UltraPureDNase/RNase-Free Distilled Water (Invitrogen, Life Technologies, Grand Island, NY, USA). These eluted samples were kept on ice and vortexed five times for 10 s each. This process was repeated for 20 min. RNA was recovered from the FTA cards using the TRIzol method and was used to detect ZIKV as previously described.

### Transmission electron microscopy

Salivary glands from *Cx. quinquefasciatus* were dissected at 7 dpi and fixed for 2 h in a solution containing 2.5% glutaraldehyde and 4% paraformaldehyde in 0.1 M cacodylate buffer solution. After fixation, the samples were washed twice in the same buffer and post-fixed in a solution containing 1% osmium tetroxide, 2 mM calcium chloride and 0.8% potassium ferricyanide in 0.1 M cacodylate buffer, pH 7.2, dehydrated in acetone as previously reported,^33^ and were then embedded using a Fluka Epoxy Embedding kit (Fluka Chemie AG, Buchs, Switzerland). Polymerization was performed at 60 °C for 24 h. Ultrathin sections (70 nm) were placed on 300-mesh nickel grids and then counterstained with 5% uranyl acetate and lead citrate, followed by examination using a transmission electron microscope (Tecnai Spirit Biotwin, FEI Company, Hillsboro, OR, USA).

### Statistical analysis

The IR and SR were calculated for each species at different time points. Logistic regression, *χ*^2^-test and Fisher’s Exact tests were used to test for differences in both the IR and SR within the two species. The Cochran–Mantel–Haenszel and log linear regression tests were also performed to compare differences between the species. All statistical tests were performed with R software (R DEVELOPMENT CORE TEAM, 2012). GraphPad Prism software v.5.02 (GraphPad, San Diego, CA, USA) was used to plot graphics and to compare viral genome quantification values between the different time points, tissues and samples using an unpaired *t*-test.

### ZIKV detection and viral isolation in field-collected mosquitoes

Mosquito samples were collected in the metropolitan region of Recife, from February to May 2016, in two distinct types of locations: at premises where zika cases were noted and at public Emergency Care Units. Pernambuco Secretary of Health personnel carried out collections at Emergency Care Units, and our own fieldwork team collected mosquitoes at premises with Zika infections. Both sets of collections were performed using a battery-operated aspirator (Horst Armadilhas Ltd, São Paulo, Brazil). Samples were sent alive to the IAM where they were anesthetized at 4 °C; morphologically identified; sorted by species, locality, sex and feeding status (engorged and not engorged); pooled (1–10 individuals/pool); and preserved at −80 °C until being assayed for RNA extraction and RT-qPCR as described above. The minimum IR (MIR) for ZIKV in adults captured in the field was calculated as (the number of positive pools for ZIKV/total number of mosquitoes tested) × 1000.^34^

Positive samples were assayed for virus isolation in VERO cells as follows. In a tissue culture tube (Techno Plastic Products AG, Trasadingen, Schaffhausen, Switzerland), 1 mL of a 5 × 10^5^ cells/mL suspension in Minimum Essential Media (MEM) was seeded for 24 h to form a monolayer. After that, the MEM medium was discarded, and 1 mL of the filter homogenate (100 μL of positive homogenate+900 μL of MEM medium) was inoculated in the cells. After a 1-h incubation for virus adsorption, 1 mL of fresh medium was added to the tissue culture tubes, and they were then incubated at 37 °C in 5% CO_2_ atmosphere until the detection of cytopathic effects. After that, samples were frozen and thawed twice, followed by centrifugation for 10 min at 2000 r/min. The supernatants were then transferred to cryotubes and stocked at −80 °C until further usage.

### Genome sequencing

Two positive field-collected samples of *Cx. quinquefasciatus* (Cx5 and Cx17) identified previously by RT-qPCR were inoculated in VERO cells for virus replication to obtain ZIKV first passage. For genome sequencing, total RNA from each sample was extracted from the VERO cell cultures using TRIzol reagent as described above. A MiSeq (Illumina, San Diego, CA, USA) sequencing library was prepared with a Nextera XT Library Prep Kit (Illumina) using 2 ng of input cDNA from multiplex PCR following the manufacturer's instructions. To obtain cDNA, RT-PCR reactions were carried out in a volume of 25 μL using a SuperScript III Platinum One-Step RT-qPCR Kit (Invitrogen, Life Technologies) with modifications. Briefly, the cDNA first strand was reverse-transcribed from previously extracted RNA (minimum 10 ng for reaction) at 50 °C for 30 min, and random hexamers (random sequence [d(N)6]) were used as the first-strand primer. After reverse transcription, a PCR program was performed to amplify the whole ZIKV genome. Primers for ZIKV genome amplification were described by the ZIBRA project.^35^ A total of 35 cycles of 95 °C for 5 s and 65 °C for 15 min were performed on a Veriti Thermal Cycler (Invitrogen, Life Technologies). As described by the ZIBRA protocol, two different sets of primers were designed for a multiplex PCR, and amplification reactions from each primer set were carried out separately on single samples. After amplification, the PCR products were quantified using a Qubit dsDNA HS Assay Kit (Invitrogen, Life Technologies). A MiSeq Reagent Kit V3 of 150 cycles was used to sequence both samples using a paired-end strategy, which is expected to result in 75-bp reads separated by ~350 bp. Sequencing was performed on the MiSeq (Illumina) platform at the Technological Platform Core at the IAM.

### Reads quality check and mapping

Raw reads were processed by Trimmomatic v 0.35^36^ for adapter and low-quality read removal. FASTQC (http://www.bioinformatics.babraham.ac.uk/projects/fastqc/) was used to check Trimmomatic output, and Bowtie 2^37^ was used to map the reads against the ZIKV BRPE243/2015 reference genome (KX197192).^28^

### Spatial analysis

We georeferenced the points from which the mosquitoes were collected using WGS-84 (World Geodesic System), a GPS receiver device that processed the data in QGIS software. We generated a geographical database and performed a kernel density analysis based on the spatial distribution of reported cases of microcephaly registered by the Health Department of Pernambuco. The illustrated map shows an overlay between the location of the mosquito sampling and the kernel density map of reported cases of microcephaly from August 2015 to March 2016.

## RESULTS

### Vector competence assays

A total of 289 mosquitoes were examined for ZIKV infection by RT-qPCR. Among these mosquitoes, 130 were *Ae. aegypti* RecLab, 60 were *Ae. aegypti* Fernando de Noronha (FN) and 99 were *Cx. quinquefasciatus*.

Analysis suggests that, in general, all strains (*Ae. aegypti* RecLab, *Ae. aegypti* FN and *Cx. quinquefasciatus*) are capable of transmitting ZIKV when artificially blood fed with ZIKV. Comparing the lab strains, at the higher virus titer, the IR and SR determined for both species were similar (IR, *P*=0.2270; SR, *P*=1.0000; Table 1). We also observed that at 7 dpi both species presented their highest IR and SR. When a 10^4^ PFU/mL viral dose was administered to these laboratory strains, lower IRs and SRs were observed for both species, apart from the IR of *Ae. aegypti* at 15 dpi and its SR, which increased in the shortest extrinsic incubation period (3 dpi). Overall, at 7 dpi, the two species did not differ significantly for both the IR (*P*=0.5590) and SR (*P*=1.0000), with the exception of the SR at 10^6^ PFU/mL (*P*=0.0100; Table 1). Regarding the field-collected strain, *Ae. aegypti* FN, there was no difference between the IR for the low and high viral doses, but the difference for the SR was statistically significant (Table 2). We also sampled mosquitoes at 11 dpi in the first trial, except for field-collected *Ae. aegypti* (FN), and, although no IR was detected, a 10% SR was observed at this particular dpi. Neither *Cx. quinquefasciatus* nor *Ae. aegypti* RecLab was ZIKV-positive at 11 dpi (data not shown).

**Figure 1:**
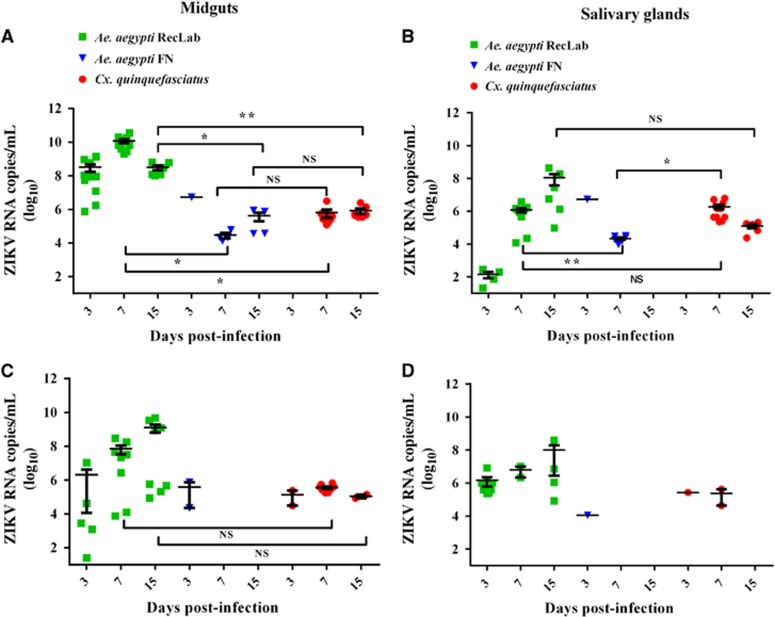
Quantification of RNA viral copy numbers in the midguts and salivary glands of *Ae. aegypti* and *Cx. quinquefasciatus* mosquitoes experimentally fed with blood containing ZIKV at 10^6^ PFU/mL (**A** and **B**) and 10^4^ PFU/mL (**C** and **D**). Green squares represent the *Ae. aegypti* (RecLab) population, blue inverted triangles represent the *Ae. aegypti* field population (FN) and red circles represent *Cx. quinquefasciatus*. Statistical analysis was performed using GraphPad Prism software (GraphPad) by unpaired *t*-test (*P<0.05 and **P<0.01). Nonsignificant, NS; Zika virus, ZIKV.

**Figure 2:**
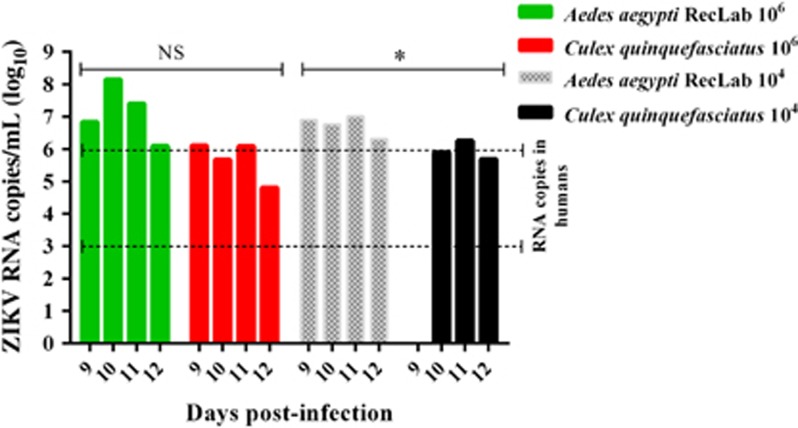
Quantification of ZIKV in *Ae. aegypti* and *Cx. quinquefasciatus* saliva expectorated onto FTA cards 9–12 days post infection (dpi). Green and red bars show the *Ae. aegypti* (RecLab) and *Cx. quinquefasciatus* populations blood-fed with ZIKV at 10^6^ PFU/mL, respectively. The hashed pattern gray and black bars show the *Ae. aegypti* (RecLab) and *Cx. quinquefasciatus* populations, respectively, blood-fed with ZIKV at 10^4^ PFU/mL. Parallel dashed lines indicate variations of ZIKV viremia in humans. Statistical analysis was performed using GraphPad Prism software (GraphPad) by unpaired *t*-test (*P<0.05). Nonsignificant, NS; Zika virus, ZIKV.

RT-qPCR was used to quantify ZIKV RNA load at 3, 7 and 15 dpi. In general, viral RNA copies in *Ae. aegypti* RecLab varied considerably in the midguts and salivary glands (Figures 1A–1D). Viral copies in the target organs (midguts and salivary glands) of both *Ae. aegypti* FN and *Cx. quinquefasciatus* remained detectable at most of the studied time points (Figures 1A–1D). To confirm ZIKV-infective particles in salivary glands, two *Ae. aegypti* RecLab and two *Cx. quinquefasciatus-*positive samples collected at 7 dpi were inoculated in VERO cells for 10 days. After that, RNA was extracted and RT-qPCR was performed; the Cts of the *Ae. aegypti* and *Cx. quinquefasciatus*-positive pools from their first passage in VERO cells when screened by RT-qPCR were 34.0. To evaluate ZIKV transmission in saliva for both species, honey-soaked filter papers (FTA Classic Cards, Whatman) were placed on the top of each cage containing mosquitoes to feed upon from 8 to 14 dpi. At 9–12 dpi, ZIKV RNA was detected in the saliva of both *Ae. aegypti* and *Cx. quinquefasciatus* species (Figure 2). When a high viral dose (10^6^) was used, the amount of viral RNA copies expectorated during salivation in both *Ae. aegypti* and *Cx. quinquefasciatus* were similar at all time points analyzed (*P*>0.05). However, when the mosquitoes were challenged with a low viral dose (10^4^), *Ae. aegypti* expectorated more RNA viral copies than *Cx. quinquefasciatus* (*P*=0.0473). It is important to highlight that we have no information regarding the number of mosquitoes that expectorated virus on the FTA cards per cage. The number of mosquitoes that salivated on the cards could affect the quantities of ZIKV particles detected by RT-qPCR.

### Transmission electron microscopy

**Figure 3:**
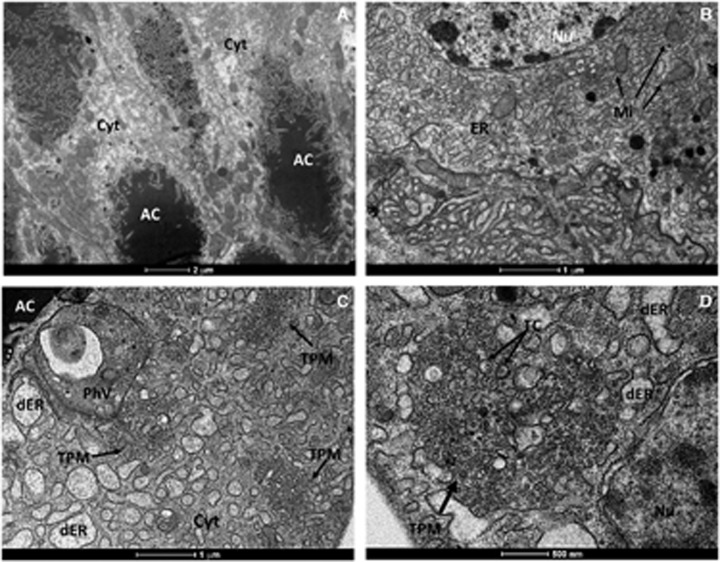
(**A** and **B**) Ultrathin sections of an uninfected *Cx. quinquefasciatus* salivary gland. (**A**) This micrograph shows the electrodense content of the apical cavity (**A** and **C**) with membrane projections that extend from the wall. (**B**) Uninfected acinar salivary gland cell showing the Nu, ER and Mi. (**C** and **D**) Cytopathic effects of salivary glands cells infected with ZIKV showing several patches of TPM, dER and a PhV. Cell cytoplasm, Cyt; distended endoplasmic reticula, dER; endoplasmic reticulum, ER; mitochondria, Mi; nucleus, Nu; phagolysosome-like vacuole, PhV; thread-like center, TC; tubular proliferated membrane, TPM; Zika virus, ZIKV.

**Figure 4:**
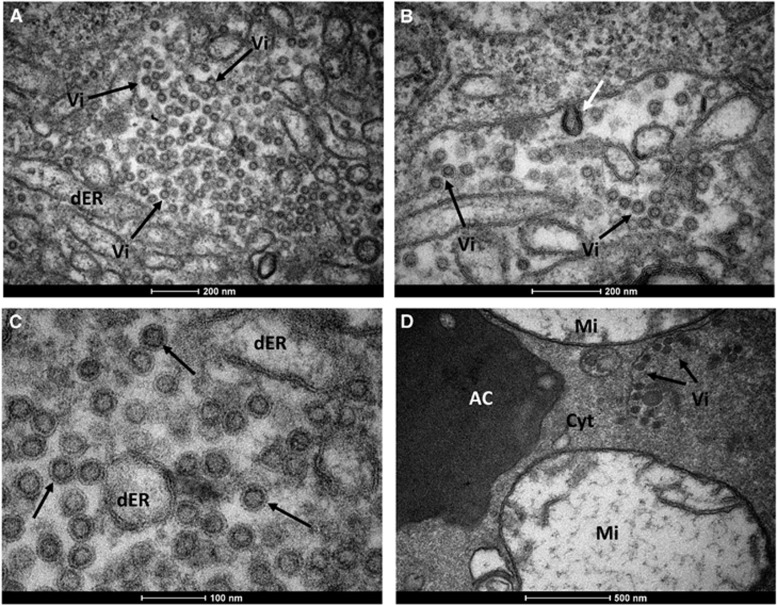
Mature ZIKV particles inside a *Cx. quinquefasciatus* salivary gland cell. (**A**) Numerous ZIKV particles (black arrows) within the dER. (**B**) Envelope formation from the endoplasmic membrane (white arrow). (**C**) Enveloped virus particles with electrodense cores. (**D**) Viral particles accumulated proximal to the acinar cavity (arrows); note the damaged mitochondria. Acinar cavity, AC; cell cytoplasm, Cyt; distended endoplasmic reticulum, dER; mitochondria, Mi; virion (s), Vi; Zika virus, ZIKV.

To further scrutinize our results from RT-qPCR, we performed transmission electron microscopy of salivary glands dissected from *Cx. quinquefasciatus* mosquitoes. The morphological organization of *Cx. quinquefasciatus* salivary glands showed an electron-dense apical cavity with membrane projections extending from the wall (Figure 3A) in addition to a basal nucleus, mitochondria and abundant endoplasmic reticulum (Figure 3B). ZIKV-infected salivary acinar cells of *Cx. quinquefasciatus* showed signs of cytopathic disruptions, including cisternae distension of the endoplasmic reticulum and tubular proliferated membranes organized in several patches within a single cell, presenting thread-like centers (Figures 3C and 3D). Mature ZIKV particles of 40–50 nm in diameter, composed of a central electrodense core (~30 nm in diameter) surrounded by a viral envelope, were observed inside the dilated endoplasmic reticulum (Figures 4A–4D). In some regions, viral envelope formation was shown to arise from the endoplasmic membrane (Figure 4B). Some ZIKV particles were observed proximal to the apical cavity of the salivary cell. Mitochondria also showed severe damage, including complete loss of cristae (Figure 4D). In summary, transmission electron microscopy analysis confirmed that *Cx. quinquefasciatus* mosquitoes are susceptible to ZIKV infection because viral particles were detected in the salivary glands of artificially fed mosquitoes.

### ZIKV detection in field-caught *Cx. quinquefasciatus*

**Figure 5:**
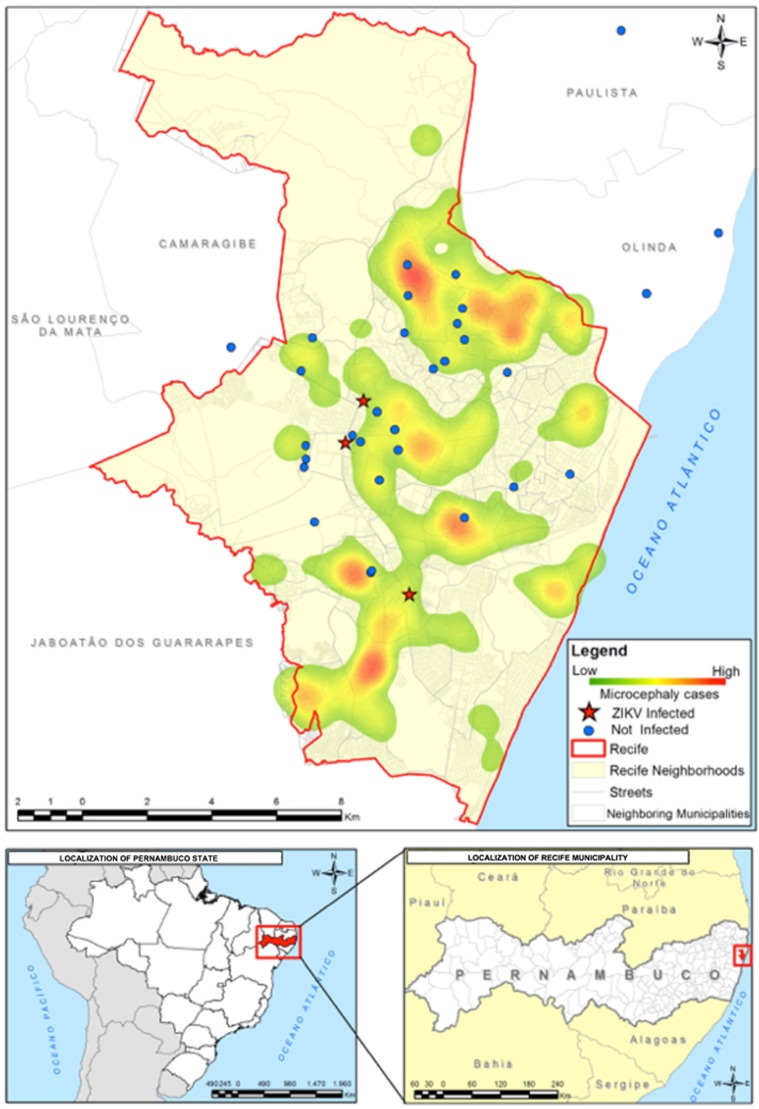
Kernel density map of reported cases of microcephaly versus a Point Map of the mosquito collection sites (with *Culex* samples positive and negative for the presence of ZIKV). Zika virus, ZIKV.

Lastly, we conducted ZIKV surveillance (February to May 2016) in mosquitoes collected from residences inhabited by individuals with clinical symptoms of Zika fever. A total of 1496 adult *Cx. quinquefasciatus* and 408 *Ae. aegypti* female mosquitoes were collected from different sites in the Metropolitan Region of Recife (Figure 5). From 270 pooled samples of adult female *Cx. quinquefasciatus* and 117 pools of *Ae. aegypti* mosquitoes assayed by RT-qPCR, three *Cx. quinquefasciatus* and two *Ae. aegypti* pools were positive for ZIKV. Interestingly, colorimetric analysis indicated no traces of undigested blood in two of the three positive *Cx. quinquefasciatus* samples and undigested blood in the two *Ae. aegypti*-positive pools. These findings suggest that, at least in the two ZIKV-positive *Cx. quinquefasciatus* samples, the virus was not derived from a recent blood meal. The Cts of the *Cx. quinquefasciatus*-positive pools when screened by RT-qPCR were 37.6 (sample 5), 38.0 (sample 17) and 38.15 (sample 163). For the *Ae. aegypti* pools, the Cts were 37.5 (sample 3) and 37.9 (sample 7). The MIR was calculated for both species; for *Cx. quinquefasciatus*, the MIR was 2.0‰ and for *Ae. aegypti* the MIR was 4.9‰. In an attempt to isolate ZIKV from the field-caught *Culex* mosquitoes, we inoculated African Green Monkey kidney cells (VERO cells) with samples from the two positive pools with the lowest Cts. ZIKV was isolated from these samples, thus unambiguously demonstrating that this species was carrying active ZIKV particles in Recife, Brazil.

### ZIKV genome sequencing from field-caught *Cx. quinquefasciatus*

ZIKV was obtained from the first passage of two pools of field-caught *Cx. quinquefasciatus* in VERO cells. These viruses were named ZIKV/*C. quinquefasciatus*/Brazil/PE05/2016 (Cx5) and ZIKV/*C. quinquefasciatus*/Brazil/PE17/2016 (Cx17). We obtained two draft ZIKV genomes from these samples that were 5365 (Cx5) and 5380 (Cx17) nucleotides long, covering more than 50% of the virus genome at a coverage depth average of 13.32 and 12.18 ×, respectively. The two partial genomes were deposited in GenBank under accession numbers KX986760 and KX986761, respectively.

Sequence alignment of ZIKV PE243/2015 with the Cx5 partial genome detected a single-nucleotide polymorphism (SNP) in the ZIKV envelope region of the mosquito sample. A comparison between PE243 and Cx17 showed the presence of 10 different SNPs in the partial ZIKV genome from Cx17. Six and four SNPs were found in the NS3 and NS5 regions, respectively. The first five SNPs found in the NS3 region were supported by 11 reads, while the last one was supported by five reads. All four SNPs observed in the NS5 region were supported by seven reads.

## DISCUSSION

Our work has associated a second mosquito genus with the ZIKV transmission cycle in northeast Brazil. We showed that *Cx. quinquefasciatus*, also known as the southern house mosquito, which is the most common mosquito in urban areas in Brazil, is susceptible to infection with ZIKV during experimental blood feeding; moreover, we found that ZIKV has an active replication cycle in the salivary glands and is subsequently released in the saliva. In addition, we were able to detect ZIKV circulating in wild *Cx. quinquefasciatus* collected from Recife, and, for the first time, we sequenced a partial region of a ZIKV genome (50%) obtained from mosquito samples in Brazil.

Although *Ae. aegypti* is widely assumed to be the main ZIKV vector, previous vector competence studies are controversial. In the present study, a low dose of ZIKV (10^4^ PFU/mL) was used for comparison with the higher doses used in previous studies.^11, 12, 15^ We found that both *Ae. aegypti* and *Cx. quinquefasciatus* can be experimentally infected by ZIKV even at low doses and can subsequently expectorate ZIKV in their saliva.

Cornet *et al.*^10^ concluded that part of a population of infected mosquitoes may not be able to transmit the virus, and that, even if they do, transmission occurs during a particular timeframe. This hypothesis differs from the idea that, once infected, a mosquito would transmit virus for its entire life. This finding has implications for arbovirus surveillance programs as a time window for vector-borne ZIKV transmission may exist. We found that after 11 dpi, most samples were negative for ZIKV (apart from one positive salivary gland of *Ae. aegypti* fed 10^6^ PFU/mL); thus, our maximum time point analysis was set to 15 dpi. However, Boorman and Porterfield^8^ reported that virus replication resumed at 15–20 dpi, and ZIKV remained present in *Aedes* mosquitoes for up to 60 days.

To confirm that the virus detected in the salivary glands by RT-qPCR was being released in saliva during consecutive blood meals, we followed the viral load from days 8 to 14 post infection using filter paper cards. FTA cards were always placed on the top of the cages to avoid excreta contamination as noted in previous studies.^38, 39^ This strategy of viral RNA detection directly from FTA cards has been employed in previous studies for arbovirus surveillance.^40, 41^ In the present study, we successfully detected ZIKV RNA copies in cards from *Ae. aegypti* and *Cx. quinquefasciatus* mosquitoes. This result demonstrates that, in addition to being susceptible to ZIKV infection and allowing virus replication in the salivary glands, both species are capable of effectively transmitting ZIKV.

RT-qPCR results were confirmed by transmission electron microscopy. The general mature ZIKV morphology observed for the salivary glands confirmed previous ultrastructural studies.^42, 43, 44^ In salivary gland cells, ZIKV replication causes cytopathic effects by 7 dpi, such as tubular membrane proliferation, similar to results shown elsewhere for West Nile virus.^45, 46^ The fact that we found salivary glands positive for ZIKV when the midgut of the same mosquito was negative indicates that mosquitoes may be clearing the viral infection in the midgut while virus replication continues in the salivary glands. This finding has implications for the analytical methods employed in vector competence studies.

It is interesting to note that we were able not only to demonstrate ZIKV infection in *Cx. quinquefasciatus* in laboratory assays but also in field-caught mosquitoes. Viruses isolated from field-caught *Cx. quinquefasciatus* were passed in VERO cells and sequenced, and mutations in the NS3 and NS5 regions were detected. These particular regions encompass an RNA helicase (NS3-Hel) and a putative RNA-dependent RNA polymerase (NS5-RdRp), which are key components for genome replication and RNA synthesis.^47^ In addition, more notably, these polymorphisms were not identified in the ZIKV strain circulating in Recife (PE243) that was obtained from human sera. Our findings are supported by Guo *et al.*,^23^ but we are cognizant of previous studies that were unable to confirm ZIKV replication in *Cx. quinquefasciatus*.^17, 18, 21, 24, 48^

Early studies have targeted only *Aedes* species, and only recent studies have compared the natural IRs of different species (including *Culex*). Notably, Diallo *et al.*^5^ observed a higher MIR for *Cx. perfuscus* (10 × higher) than for *Ae. aegypti*. Positive *Ae. aegypti* field samples have always been reported at very low IRs, even in areas with high human ZIKV IRs, such as Malaysia.^49^ Indeed, in Micronesia^6^ and French Polynesia, ZIKV was not detected in wild-caught *Aedes* spp. mosquitoes during outbreaks. It is interesting that in all of these areas, *Cx. quinquefasciatus* is an abundant mosquito species that may have also had an undetected role in ZIKV transmission. Indeed, Richard *et al.*^13^ demonstrated that two *Aedes* species from French Polynesia did not show enough vector competence to transmit ZIKV and suggested that another mosquito species could be contributing to the spread of the virus in that region.

Thus, our findings indicate that vector control strategies may need to be re-examined since reducing *Ae. aegypti* populations may not lead to an overall reduction in ZIKV transmission if *Culex* populations are not affected by *Aedes-*specific control measures. At the moment, there is no broad ongoing program for *Cx. quinquefasciatus* control in Brazil, although Recife, Olinda and Jaboatão dos Guararapes, three municipalities in the Recife Metropolitan Region, have undertaken specific control of *Cx. quinquefasciatus* to control lymphatic filariasis (LF) transmission locally,^50^ as this species is the only LF vector.

Viral transmission via *Cx. quinquefasciatus* is not a new concept; in North America, this species is the major vector of West Nile virus^51^ and it is also a known vector of Japanese encephalitis virus^52^ and equine encephalitis virus.^53^ Our data indicate that *Cx. quinquefasciatus* mosquitoes may be involved in ZIKV transmission in Recife. Thus, understanding the contributions of each species in the transmission of ZIKV is necessary to target each one properly. In conclusion, considering its high abundance in urban environments and its anthropophilic behavior in Brazil,^54, 55, 56^ *Cx. quinquefasciatus* may be a vector for ZIKV in this region.

## Figures and Tables

**Table 1:** IR and proportion of infected SR of *Aedes aegypti* and *Culex quinquefasciatus* laboratory colonies after artificial blood feeding with ZIKV American strain (ZIKV BRPE243/2015)

**Dose**	**Day**	***Aedes aegypti*** **(RecLab)**				***Culex quinquefasciatus***				**Total**				**Among species; *****P*****-value**
		**+**		**−**		**+**		**−**		**+**		**−**		
		***N***	**%**	***N***	**%**	***N***	**%**	***N***	**%**	***N***	**%**	***N***	**%**	
10 ^6^ PFU/mL														
IR	3	14	77.78	4	22.22					14	77.78	4	22.22	
	7	18	90.00	2	10.00	10	83.33	2	16.67	28	87.50	4	12.50	0.6196
	15	7	43.75	9	56.25	7	38.89	11	61.11	14	41.18	20	58.82	1.0000
	Total	39	72.22	15	27.78	17	56.67	13	43.33					
	*P*-value	0.0091				0.0256								
										***P*-value**				
										**Species**		**Day**		
										0.2270		0.0002		

SR	3	4	20.00	16	80.00					4	20.00	16	80.00	
	7	12	60.00	8	40.00	12	100.00	0	0.00	24	75.00	8	25.00	0.0100
	15	6	37.50	10	62.50	5	27.78	13	72.22	11	32.35	23	67.65	0.7170
	Total	22	59.46	15	40.54	17	56.67	13	43.33					
	*P*-value	0.0388				<0.0001								
										***P*-value**				
										**Species**		**Day**		
										1.0000		<0.0001		

10 ^4^ PFU/mL														
IR	3	8	40.00	12	60.00	2	20.00	8	80.00	10	33.33	20	66.67	0.4190
	7	9	45.00	11	55.00	9	36.00	16	64.00	18	40.00	27	60.00	0.5590
	15	9	50.00	9	50.00	2	10.53	17	89.47	11	29.73	26	70.27	0.0128
	Total	26	44.83	32	55.17	13	24.07	41	75.93					
	*P*-value	0.9443				0.1762								
										***P*-value**				
										**Species**		**Day**		
										0.0353		0.6120		

SR	3	11	55.00	9	45.00	1	10.00	9	90.00	12	40.00	18	60.00	0.0235
	7	2	10.00	18	90.00	2	8.00	23	92.00	4	8.89	41	91.11	1.0000
	15	4	22.22	14	77.78	0	0.00	19	100.00	4	10.81	33	89.19	0.0463
	Total	17	29.31	41	70.69	3	5.56	51	94.44					
	*P*-value	0.0056				0.4063								
										***P*-value**				
										**Species**		**Day**		
										0.0024		0.0010		
												
*P*-value	Between IR	0.0750				0.0014								
	Between SR	0.4702				<0.0001								

Abbreviations: infection rate, IR; proportion of infected salivary gland, SR; Zika virus, ZIKV.

‘*N*’ represents the number of analyzed individuals. Statistical analyses were performed using the R package (R DEVELOPMENT CORE TEAM, 2012). The significance level was set at *P*<0.05.

**Table 2:** IR and SR of the *Aedes aegypti* field-caught (Fernando de Noronha—FN) colony after artificial blood feeding with ZIKV American strain (ZIKV BRPE243/2015)

		***Aedes aegypti*** **(FN)**								***P*****-value**			
		**IR**				**SR**							
**Dose**	**Day**	**+**		**−**		**+**		**−**		**IR**	SR	**Between dose**	
		***N***	**%**	***N***	**%**	***N***	**%**	***N***	**%**			**IR**	**SR**
10 ^6^ PFU/mL	3	1	10.00	9	90.00	1	10.00	9	90.00	0.2967	0.0064	0.3485	0.0018
	7	4	40.00	6	60.00	6	60.00	4	40.00				
	15	4	40.00	6	60.00	0	0.00	10	100.00				
	Total	9	30.00	21	70.00	7	25.00	21	75.00				
10 ^4^ PFU/mL	3	2	20.00	8	80.00	1	10.00	9	90.00	0.4737	1.0000		
	7	0	0.00	10	100.00	0	0.00	10	100.00				
	15												
	Total	2	10.00	18	90.00	1	5.00	19	95.00				

Abbreviations: infection rate, IR; proportion of infected salivary gland, SR; Zika virus, ZIKV.

‘*N*’ represents the number of analyzed individuals. Statistical analyses were performed using the R package (R DEVELOPMENT CORE TEAM, 2012). The significance level was set at *P*<0.05.

## References

[bib1] Chang C, Ortiz K, Ansari A et al. The Zika outbreak of the 21st century. J Autoimmun 2016; 68: 1–13. 26925496 10.1016/j.jaut.2016.02.006 PMC7127657

[bib2] Cordeiro MT, Pena LJ, Brito CA et al. Positive IgM for Zika virus in the cerebrospinal fluid of 30 neonates with microcephaly in Brazil. Lancet 2016; 387: 1811–1812. 10.1016/S0140-6736(16)30253-7 27103126

[bib3] Moron AF, Cavalheiro S, Milani H et al. Microcephaly associated with maternal Zika virus infection. BJOG 2016; 123: 1265–1269. 27150580 10.1111/1471-0528.14072

[bib4] Dick GW, Kitchen SF, Haddow AJ. Zika virus. I. Isolations and serological specificity. Trans R Soc Trop Med Hyg 1952; 46: 509–520. 12995440 10.1016/0035-9203(52)90042-4

[bib5] Diallo D, Sall AA, Diagne CT et al. Zika virus emergence in mosquitoes in southeastern Senegal, 2011. PLoS ONE 2014; 9: e109442. 25310102 10.1371/journal.pone.0109442 PMC4195678

[bib6] Duffy MR, Chen TH, Hancock WT et al. Zika virus outbreak on Yap Island, Federated States of Micronesia. N Engl J Med 2009; 360: 2536–2543. 19516034 10.1056/NEJMoa0805715

[bib7] Savage HM, Fritz CL, Rutstein D et al. Epidemic of dengue-4 virus in Yap State, Federated States of Micronesia, and implication of *Aedes hensilli* as an epidemic vector. Am J Trop Med Hyg 1998; 58: 519–524. 9574802 10.4269/ajtmh.1998.58.519

[bib8] Boorman JP, Porterfield JS. A simple technique for infection of mosquitoes with viruses; transmission of Zika virus. Trans R Soc Trop Med Hyg 1956; 50: 238–242. 13337908 10.1016/0035-9203(56)90029-3

[bib9] Li MI, Wong PS, Ng LC et al. Oral susceptibility of Singapore *Aedes* (Stegomyia) *aegypti* (Linnaeus) to Zika virus. PLoS Negl Trop Dis 2012; 6: e1792. 22953014 10.1371/journal.pntd.0001792 PMC3429392

[bib10] Cornet M, Robin Y, Adam C et al. Comparison between experimental transmission of yellow fever and zika viruses in *Aedes aegypti*. Cah Orstom 1979; 17: 7.

[bib11] Dutra HL, Rocha MN, Dias FB et al. Wolbachia blocks currently circulating Zika virus isolates in Brazilian *Aedes aegypti* mosquitoes. Cell Host Microbe 2016; 19: 771–774. 27156023 10.1016/j.chom.2016.04.021 PMC4906366

[bib12] Chouin-Carneiro T, Vega-Rua A, Vazeille M et al. Differential susceptibilities of *Aedes aegypti* and *Aedes albopictus* from the Americas to Zika virus. PLoS Negl Trop Dis 2016; 10: e0004543. 26938868 10.1371/journal.pntd.0004543 PMC4777396

[bib13] Richard V, Paoaafaite T, Cao-Lormeau VM. Vector competence of French Polynesian *Aedes aegypti* and *Aedes polynesiensis* for Zika virus. PLoS Negl Trop Dis 2016; 10: e0005024. 27654962 10.1371/journal.pntd.0005024 PMC5031459

[bib14] Bearcroft WG. Zika virus infection experimentally induced in a human volunteer. Trans R Soc Trop Med Hyg 1956; 50: 442–448. 13380987

[bib15] Diagne CT, Diallo D, Faye O et al. Potential of selected Senegalese *Aedes* spp. mosquitoes (Diptera: Culicidae) to transmit Zika virus. BMC Infect Dis 2015; 15: 492. 26527535 10.1186/s12879-015-1231-2 PMC4629289

[bib16] Ayres CF. Identification of Zika virus vectors and implications for control. Lancet Infect Dis 2016; 16: 278–279. 26852727 10.1016/S1473-3099(16)00073-6

[bib17] Aliota MT, Peinado SA, Osorio JE et al. *Culex pipiens* and *Aedes triseriatus* mosquito susceptibility to Zika virus. Emerg Infect Dis 2016; 22: 1857–1859. 27434194 10.3201/eid2210.161082 PMC5038408

[bib18] Fernandes RS, Campos SS, Ferreira-de-Brito A et al. *Culex quinquefasciatus* from Rio de Janeiro Is not competent to transmit the local Zika virus. PLoS Negl Trop Dis 2016; 10: e0004993. 27598421 10.1371/journal.pntd.0004993 PMC5012671

[bib19] Hall-Mendelin S, Pyke AT, Moore PR et al. Assessment of local mosquito species incriminates *Aedes aegypti* as the potential vector of Zika virus in Australia. PLoS Negl Trop Dis 2016; 10: e0004959. 27643685 10.1371/journal.pntd.0004959 PMC5028067

[bib20] Huang YJ, Ayers VB, Lyons AC et al. *Culex* species mosquitoes and Zika virus. Vector Borne Zoonotic Dis 2016; 16: 673–676. 27556838 10.1089/vbz.2016.2058

[bib21] Amraoui F, Atyame-Nten C, Vega-Rua A et al. *Culex* mosquitoes are experimentally unable to transmit Zika virus. Euro Surveill 2016; 21. 10.2807/1560-7917.ES.2016.21.35.30333 PMC5015461 27605159

[bib22] Ferreira-de-Brito A, Ribeiro IP, Miranda RM et al. First detection of natural infection of *Aedes aegypti* with Zika virus in Brazil and throughout South America. Mem Inst Oswaldo Cruz 2016; 111: 655–658. 27706382 10.1590/0074-02760160332 PMC5066335

[bib23] Guo XX, Li CX, Deng YQ et al. *Culex pipiens quinquefasciatus*: a potential vector to transmit Zika virus. Emerg Microbes Infect 2016; 5: e102. 27599470 10.1038/emi.2016.102 PMC5113053

[bib24] Weger-Lucarelli J, Ruckert C, Chotiwan N et al. Vector competence of American mosquitoes for three strains of Zika virus. PLoS Negl Trop Dis 2016; 10: e0005101. 27783679 10.1371/journal.pntd.0005101 PMC5081193

[bib25] Leal W. Zika mosquito vectors: the jury is still out [version 2; referees: 5 approved]. F1000Research 2016; 5: 2546–2556. 27853521 10.12688/f1000research.9839.1 PMC5105876

[bib26] Amorim LB, Helvecio E, de Oliveira CM et al. Susceptibility status of *Culex quinquefasciatus* (Diptera: Culicidae) populations to the chemical insecticide temephos in Pernambuco, Brazil. Pest Manag Sci 2013; 69: 1307–1314. 23576326 10.1002/ps.3502

[bib27] Araujo AP, Melo-Santos MA, Carlos SO et al. Evaluation of an experimental product based on Bacillus thuringiensis sorovar. israelensis against Aedes aegypti larvae (Diptera: Culicidae). Biol Control 2017; 41: 339–347.

[bib28] Donald CL, Brennan B, Cumberworth SL et al. Full genome sequence and sfRNA interferon antagonist activity of Zika VIRUS from Recife, Brazil. PLoS Negl Trop Dis 2016; 10: e0005048. 27706161 10.1371/journal.pntd.0005048 PMC5051680

[bib29] Scheuermann RH. Zika virus: designate standardized names. Nature 2016; 531: 173. 10.1038/531173a 26961650

[bib30] Salazar MI, Richardson JH, Sanchez-Vargas I et al. Dengue virus type 2: replication and tropisms in orally infected *Aedes aegypti* mosquitoes. BMC Microbiol 2007; 7: 9. 17263893 10.1186/1471-2180-7-9 PMC1797809

[bib31] Lambrechts L, Chevillon C, Albright RG et al. Genetic specificity and potential for local adaptation between dengue viruses and mosquito vectors. BMC Evol Biol 2009; 9: 160. 19589156 10.1186/1471-2148-9-160 PMC2714696

[bib32] Lanciotti RS, Kosoy OL, Laven JJ et al. Genetic and serologic properties of Zika virus associated with an epidemic, Yap State, Micronesia, 2007. Emerg Infect Dis 2008; 14: 1232–1239. 18680646 10.3201/eid1408.080287 PMC2600394

[bib33] Donato MA, Ribeiro EL, Torres Dde O et al. Chronic treatment with Sildenafil has no effect on folliculogenesis or fertility in C57BL/6 and C57BL/6 knockout for iNOS mice. Tissue Cell 2015; 47: 515–525. 26250484 10.1016/j.tice.2015.07.001

[bib34] Chow VT, Chan YC, Yong R et al. Monitoring of dengue viruses in field-caught *Aedes aegypti* and *Aedes albopictus* mosquitoes by a type-specific polymerase chain reaction and cycle sequencing. Am J Trop Med Hyg 1998; 58: 578–586. 9598444 10.4269/ajtmh.1998.58.578

[bib35] Faria NR, Sabino EC, Nunes MR et al. Mobile real-time surveillance of Zika virus in Brazil. Genome Med 2016; 8: 97. 27683027 10.1186/s13073-016-0356-2 PMC5041528

[bib36] Bolger AM, Lohse M, Usadel B. Trimmomatic: a flexible trimmer for Illumina sequence data. Bioinformatics 2014; 30: 2114–2120. 24695404 10.1093/bioinformatics/btu170 PMC4103590

[bib37] Langmead B, Salzberg SL. Fast gapped-read alignment with Bowtie 2. Nat Methods 2012; 9: 357–359. 22388286 10.1038/nmeth.1923 PMC3322381

[bib38] Fontaine A, Jiolle D, Moltini-Conclois I et al. Excretion of dengue virus RNA by *Aedes aegypti* allows non-destructive monitoring of viral dissemination in individual mosquitoes. Sci Rep 2016; 6: 24885. 27117953 10.1038/srep24885 PMC4846815

[bib39] Pilotte N, Zaky WI, Abrams BP et al. A novel xenomonitoring technique using mosquito excreta/feces for the detection of filarial parasites and malaria. PLoS Negl Trop Dis 2016; 10: e0004641. 27096156 10.1371/journal.pntd.0004641 PMC4838226

[bib40] Flies EJ, Toi C, Weinstein P et al. Converting mosquito surveillance to arbovirus surveillance with honey-baited nucleic acid preservation cards. Vector Borne Zoonotic Dis 2015; 15: 397–403. 26186511 10.1089/vbz.2014.1759

[bib41] Hall-Mendelin S, Ritchie SA, Johansen CA et al. Exploiting mosquito sugar feeding to detect mosquito-borne pathogens. Proc Natl Acad Sci USA 2010; 107: 11255–11259. 20534559 10.1073/pnas.1002040107 PMC2895145

[bib42] Bell TM, Field EJ, Narang HK. Zika virus infection of the central nervous system of mice. Arch Gesamte Virusforsch 1971; 35: 183–193. 5002906 10.1007/BF01249709

[bib43] Mlakar J, Korva M, Tul N et al. Zika virus associated with microcephaly. N Engl J Med 2016; 374: 951–958. 26862926 10.1056/NEJMoa1600651

[bib44] Rosen L. Overwintering mechanisms of mosquito-borne arboviruses in temperate climates. Am J Trop Med Hyg 1987; 37: 69S–76S. 2891312 10.4269/ajtmh.1987.37.69s

[bib45] Girard YA, Popov V, Wen J et al. Ultrastructural study of West Nile virus pathogenesis in *Culex pipiens quinquefasciatus* (Diptera: Culicidae). J Med Entomol 2005; 42: 429–444. 15962797 10.1093/jmedent/42.3.429

[bib46] Girard YA, Schneider BS, McGee CE et al. Salivary gland morphology and virus transmission during long-term cytopathologic West Nile virus infection in *Culex* mosquitoes. Am J Trop Med Hyg 2007; 76: 118–128. 17255239

[bib47] Zhu Z, Chan JF, Tee KM et al. Comparative genomic analysis of pre-epidemic and epidemic Zika virus strains for virological factors potentially associated with the rapidly expanding epidemic. Emerg Microbes Infect 2016; 5: e22. 26980239 10.1038/emi.2016.48 PMC4820678

[bib48] Boccolini D, Toma L, Di Luca M et al. Experimental investigation of the susceptibility of Italian *Culex pipiens* mosquitoes to Zika virus infection. Euro Surveill 2016; 21: e30328. 10.2807/1560-7917.ES.2016.21.35.30328 PMC5015456 27605056

[bib49] Marchette NJ, Garcia R, Rudnick A. Isolation of Zika virus from *Aedes aegypti* mosquitoes in Malaysia. Am J Trop Med Hyg 1969; 18: 411–415. 4976739 10.4269/ajtmh.1969.18.411

[bib50] Chalegre KD, Romao TP, Amorim LB et al. Detection of an allele conferring resistance to *Bacillus sphaericus* binary toxin in *Culex quinquefasciatus* populations by molecular screening. Appl Environ Microbiol 2009; 75: 1044–1049. 19098223 10.1128/AEM.02032-08 PMC2643579

[bib51] Nasci RS, White DJ, Stirling H et al. West Nile virus isolates from mosquitoes in New York and New Jersey, 1999. Emerg Infect Dis 2001; 7: 626–630. 11585523 10.3201/eid0704.010404 PMC2631761

[bib52] Sucharit S, Surathin K, Shrestha SR. Vectors of Japanese encephalitis virus (JEV): species complexes of the vectors. Southeast Asian J Trop Med Public Health 1989; 20: 611–621. 2576966

[bib53] Wang Z, Zhang X, Li C et al. Vector competence of five common mosquito species in the People's Republic of China for Western equine encephalitis virus. Vector Borne Zoonotic Dis 2012; 12: 605–608. 22276651 10.1089/vbz.2011.0660

[bib54] Nunes AT, Brito NF, Oliveira DS et al. Comparative proteome analysis reveals that blood and sugar meals induce differential protein expression in *Aedes aegypti* female heads. Proteomics 2016; 16: 2582–2586. 27343150 10.1002/pmic.201600126

[bib55] Goertz GP, Fros JJ, Miesen P et al. Non-coding subgenomic flavivirus RNA is processed by the mosquito RNAi machinery and determines West Nile virus transmission by *Culex pipiens* mosquitoes. J Virol 2016; 90: 10145–10159. 27581979 10.1128/JVI.00930-16 PMC5105652

[bib56] Regis L, Silva-Filha MH, de Oliveira CM et al. Integrated control measures against *Culex quinquefasciatus,* the vector of filariasis in Recife. Mem Inst Oswaldo Cruz 1995; 90: 115–119. 8524072 10.1590/s0074-02761995000100022

